# Protease‐Activated Plasmonic Nanosensors for Predictive Ultrasound‐Guided Photoacoustic Imaging of Tumor Responses to Adoptive T Cell Therapy

**DOI:** 10.1002/advs.202515111

**Published:** 2025-12-14

**Authors:** Myeongsoo Kim, Seoyoon Song, Ali Zamat, Paul S. Pelkowski, Shivashankar Subramanian, Melissa Cadena, Sydney Fabrega, Meredith Brienen, Jinhwan Kim, Gabriel A. Kwong, Stanislav Y. Emelianov

**Affiliations:** ^1^ Wallace H. Coulter Department of Biomedical Engineering Georgia Institute of Technology and Emory University School of Medicine Atlanta GA 30332 USA; ^2^ Petit Institute for Bioengineering and Bioscience Georgia Institute of Technology Atlanta GA 30332 USA; ^3^ Department of Biomedical Engineering University of California Davis Davis CA 95616 USA; ^4^ Department of Surgery School of Medicine University of California Davis Sacramento CA 95817 USA; ^5^ Institute for Electronics and Nanotechnology Georgia Institute of Technology Atlanta GA 30332 USA; ^6^ Integrated Cancer Research Center Georgia Institute of Technology Atlanta GA 30332 USA; ^7^ Georgia Immunoengineering Consortium Emory University and Georgia Institute of Technology Atlanta GA 30332 USA; ^8^ Winship Cancer Institute Emory University Atlanta GA 30322 USA; ^9^ School of Electrical and Computer Engineering Georgia Institute of Technology Atlanta GA 30332 USA

**Keywords:** adoptive T cell therapy, plasmon coupling, protease‐activated nanosensor, T cell activity, ultrasound‐guided photoacoustic imaging

## Abstract

Adoptive T cell therapy (ACT) is a promising strategy for cancer treatment that harnesses a patient's own T lymphocytes to enhance antitumor immunity. A major challenge in assessing therapeutic responses following ACT is the lack of robust, noninvasive tools to monitor cytotoxic T cell activity within tumors with anatomical context. Here, a protease‐activated plasmonic nanosensor is reported for noninvasive photoacoustic (PA) imaging of ACT responses. The nanosensor comprises gold nanospheres functionalized with peptide substrates of granzyme B (GzmB), a key effector protease secreted by cytotoxic T cells. Upon peptide cleavage by GzmB, nanosensor aggregation is induced, leading to plasmon coupling and enhanced optical absorption with ≈90% efficiency in the near‐infrared optical window. This aggregation significantly amplifies PA signals, enabling sensitive detection of GzmB. The nanosensor exhibits high specificity for GzmB over other proteases and correlates optical and PA responses with antigen‐specific T cell‐mediated cytotoxicity in vitro. In murine ACT models, systemic nanosensor administration enables detection of tumor‐infiltrating cytotoxic T cell activity, producing elevated PA signals in antigen‐positive tumors compared to antigen‐negative controls before any measurable differences in tumor volume. This study presents a noninvasive approach for assessing ACT efficacy via GzmB‐activated plasmonic nanosensors combined with ultrasound‐guided PA imaging.

## Graphical abstract

Protease‐activated plasmonic nanosensors are demonstrated to noninvasively monitor antitumor T cell activity in tumors with anatomical information upon adoptive T cell transfer via ultrasound‐guided photoacoustic imaging. These nanosensors are actuated by granzyme B, a protease secreted by activated cytotoxic T cells during tumor cell killing, which triggers interparticle plasmon coupling and thus amplifies optical and photoacoustic responses. Upon systemic nanosensor delivery during adoptive T cell therapy, the nanosensors enable detection of tumor‐infiltrating cytotoxic T cell activity, with enhanced photoacoustic signals correlating with therapeutic responses.

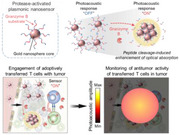

## 1 Introduction

Adoptive T cell therapy (ACT) is a clinical approach for cancer treatment in which a patient's own T lymphocytes are harnessed to drive antitumor immunity.^[^ ^1^, ^2^, ^3^, ^4^ ^]^ The therapeutic responses of ACT are primarily mediated by cytotoxic T cells that eliminate tumor cells through the release of effector molecules such as perforin and granzymes.^[^ ^5^, ^6^ ^]^ Currently, assessing therapeutic efficacy of T cell response in patients typically relies on quantification of biomarkers,^[^ ^1^, ^7^, ^8^ ^]^ such as the frequency of infused T cells,^[^ ^9^, ^10^ ^]^ levels of activation‐associated cytokines,^[^ ^11^ ^]^ and expression of T cell‐specific transgenes,^[^ ^12^, ^13^ ^]^ in peripheral blood or tumor samples. However, these approaches may not accurately reflect therapeutic responses due to biopsy sampling heterogeneity, variations associated with longitudinal monitoring, small sample sizes, and the invasive nature of repeated biopsies, all of which hinder real‐time, in situ assessment of T cell function within tumors.^[^ ^1^ ^]^ These limitations have motivated efforts to develop complementary, noninvasive methods to monitor antitumor activity of T cells, including synthetic biomarkers for distal quantification of T cell activity via urine analysis^[^ ^14^, ^15^ ^]^ as well as fluorescent^[^ ^16^ ^]^ and positron emission tomography (PET) imaging probes for visualizing T cell protease activity.^[^ ^17^, ^18^, ^19^ ^]^

Compared with urinary synthetic biomarkers,^[^ ^14^, ^15^ ^]^ fluorescence imaging, PET, magnetic resonance imaging (MRI), computed tomography (CT), and ultrasound‐guided photoacoustic (US/PA) imaging enable the monitoring of immune responses within tumors.^[^ ^16^, ^17^, ^18^, ^19^, ^20^ ^]^ Fluorescence imaging, however, suffers from limited tissue penetration and low spatial resolution.^[^ ^21^ ^]^ While PET, CT, and MRI provide deeper imaging capabilities, their use is restricted by several drawbacks: PET and CT are costly and involve ionizing radiation that hinders longitudinal monitoring, and MRI is expensive and time‐consuming due to its long acquisition times.^[^ ^21^, ^22^ ^]^ By comparison, US/PA imaging is cost‐effective, portable, and capable of providing real‐time functional and anatomical information in a nonionizing, noninvasive manner.^[^ ^23^, ^24^ ^]^ As a hybrid imaging modality, US imaging transmits sound waves and detects their echoes reflected from tissue interfaces to visualize structural and anatomical features.^[^ ^25^ ^]^ PA imaging, in contrast, relies on optical absorption of pulsed laser light by molecules within the near‐infrared (NIR) optical window (700–1100 nm) where biological tissues exhibit relatively low absorption and scattering, enabling deeper light penetration and reduced background signals.^[^ ^23^, ^24^, ^26^, ^27^, ^28^, ^29^, ^30^ ^]^ This absorption induces thermoelastic expansion, generating acoustic waves in the surrounding medium that are detected by US transducers.^[^ ^23^, ^24^, ^31^ ^]^ Endogenous substances, such as hemoglobin, are widely used to characterize vasculature and quantify oxygen saturation and hemodynamic parameters.^[^ ^32^, ^33^, ^34^, ^35^ ^]^ In addition, activity‐based nanosensors that report molecular or cellular pathways in particular diseases of interest, including pH,^[^ ^36^, ^37^ ^]^ metal ions,^[^ ^38^, ^39^ ^]^ small molecules,^[^ ^40^, ^41^, ^42^ ^]^ and proteases,^[^ ^43^, ^44^, ^45^, ^46^, ^47^, ^48^, ^49^, ^50^, ^51^ ^]^ can enhance molecular specificity of PA imaging within the NIR window. These nanosensors enable more specific visualization of disease processes, such as infection^[^ ^39^, ^45^ ^]^ and tumor metastasis.^[^ ^38^ ^]^ Building on these findings, incorporating activity nanosensors that report antitumor T cell activity into US/PA imaging could offer a promising strategy for noninvasive assessment of ACT therapeutic responses, alongside tumor anatomical information. However, most of these nanosensor systems are basally active, producing a detectable baseline signal even in the absence of target biomarkers, which may result in nonspecific signal accumulation and reduced specificity.

**Scheme 1:**
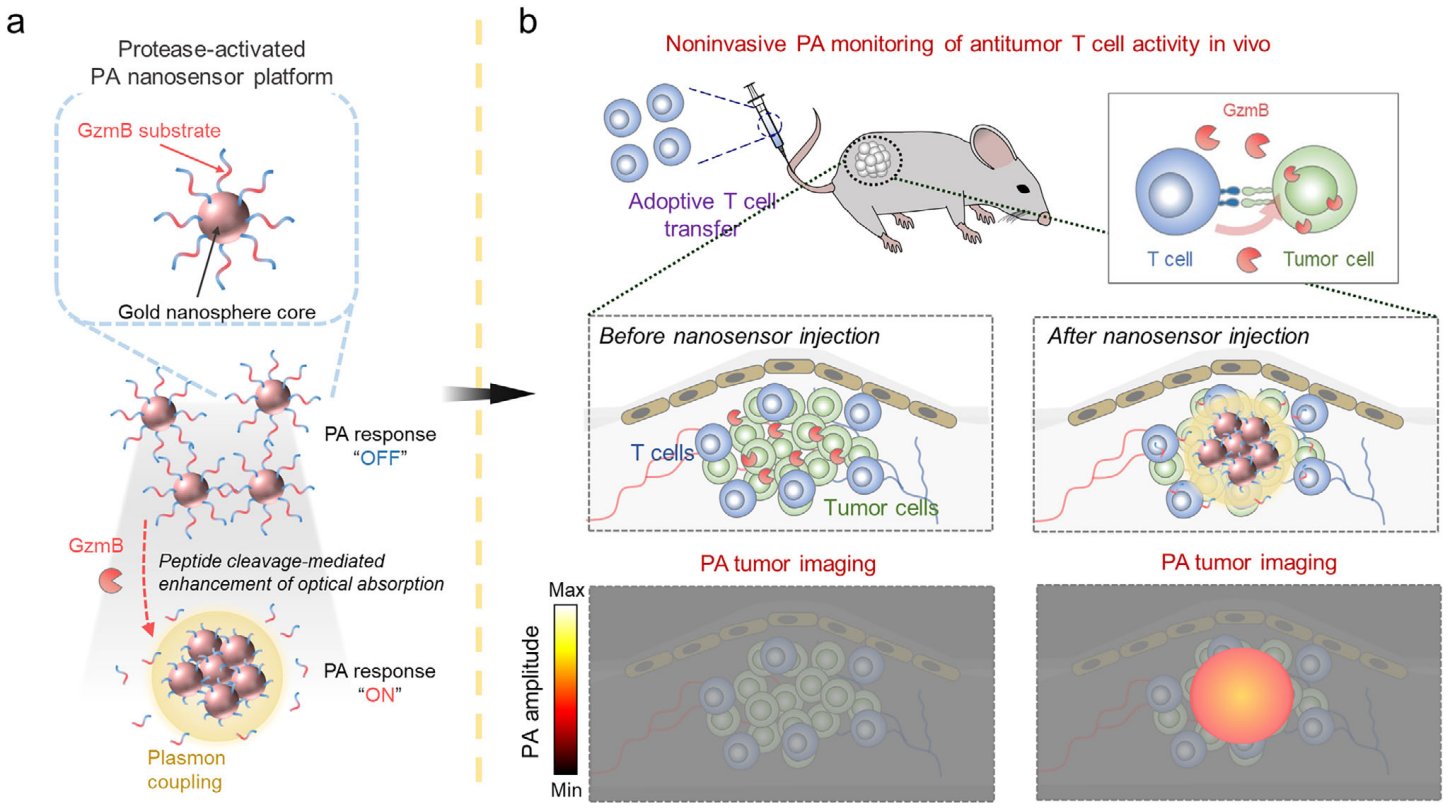
Schematic overview of the experimental approach used in this study. a) Granzyme B (GzmB)‐activated plasmonic nanosensors are composed of a gold nanosphere (GNS) core functionalized with peptides containing a GzmB‐specific cleavage sequence. Upon exposure to GzmB, these peptides are cleaved, inducing nanosensor aggregation. This aggregation enhances optical absorption and PA signals within the NIR optical window due to interparticle plasmon coupling between proximate nanosensors in the aggregate, enabling specific detection of GzmB activity via US/PA imaging within the NIR optical window. b) The GzmB‐mediated amplification of PA signals allows the nanosensors to noninvasively report the antitumor activity of tumor‐infiltrating T lymphocytes following ACT, facilitating early assessment of therapeutic response prior to observable changes in tumor burden.

In this work, we report a protease‐activated plasmonic nanosensor platform designed to exhibit a binary “OFF‐to‐ON” activation mechanism and progressive PA signal amplification for monitoring antitumor T cell activity. The nanosensors are composed of gold nanospheres (GNSs) surface‐functionalized with peptides incorporating a granzyme B (GzmB) substrate, enabling noninvasive monitoring of antitumor responses to ACT (**Scheme** **1**). GzmB, a serine protease released by activated cytotoxic T cells and associated with target cell killing,^[^ ^5^, ^14^ ^]^ serves as an imaging biomarker for evaluating ACT efficacy. Upon exposure to GzmB, cleavage of surface peptides triggers nanosensor aggregation, inducing plasmon coupling between adjacent GNSs (Scheme 1a). This coupling effect enhances optical responses in the NIR wavelength range with an absorption efficiency of ≈90%, enabling efficient PA signal conversion and amplification (Scheme 1a). The nanosensors are specifically activated by GzmB, exhibiting approximately a 7‐fold higher PA signal in response to GzmB compared to other proteases, including tumor‐associated and apoptosis‐associated proteases. Furthermore, the nanosensors detect cytotoxicity of antigen‐specific T cells in vitro, generating amplified optical and PA responses. In preclinical mouse tumor models, systemic delivery of GzmB‐activated nanosensors enables early, noninvasive evaluation of therapeutic responses to ACT via US/PA imaging, preceding measurable differences in tumor volume between antigen‐positive and antigen‐negative tumors (Scheme 1b). In addition, nanosensor‐mediated PA signal changes in tumors following ACT correlate with tumor responses. Overall, GzmB‐activated PA signal amplification from nanosensors allows them to serve as a tool for the noninvasive detection of functional T cell activity in tumors following ACT.

## 2 Results and Discussion

### 2.1 GzmB‐Activated Plasmonic Nanosensors Undergo Aggregation Upon Exposure to GzmB, Resulting in Enhanced Optical Activities in the NIR Wavelength Range

**Figure 1:**
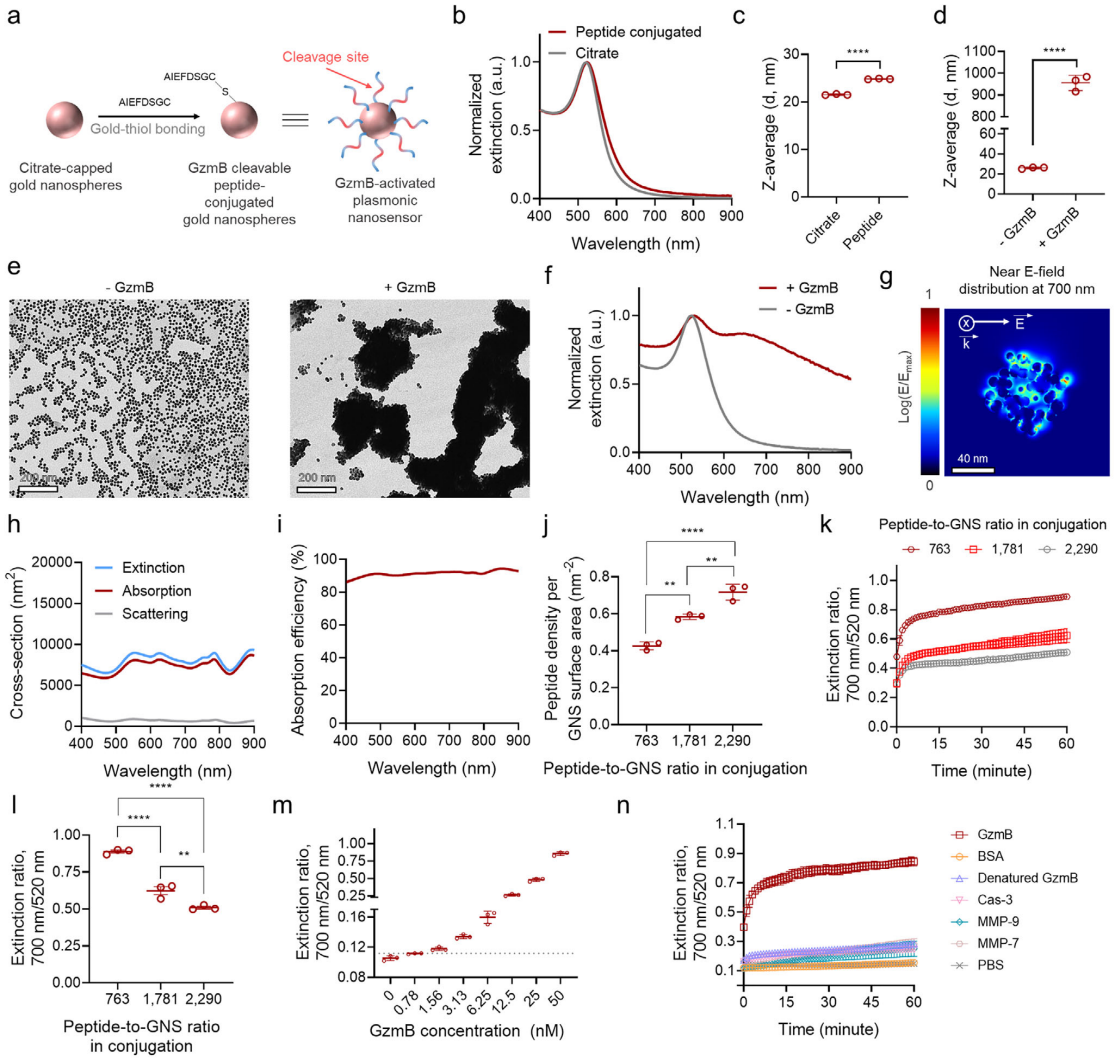
GzmB‐activated plasmonic nanosensors undergo aggregation upon exposure to GzmB, resulting in enhanced optical activities in the NIR wavelength range. a) Schematic illustration of the synthesis of GzmB‐activated plasmonic nanosensors. b,c) UV‐vis‐NIR spectra (b) and hydrodynamic diameters (c, n = 3) of GNSs before and after surface functionalization of GzmB cleavable peptides. d–f) Hydrodynamic diameters (d, n = 3), TEM images (e), and UV‐vis‐NIR spectra (f) of GzmB‐activated nanosensors before and after the addition of GzmB. g) The near electric field distribution in aggregated GNSs at a wavelength of 700 nm. h,i) Calculated optical extinction, absorption, scattering cross‐sections (h) and corresponding absorption efficiency (i) of aggregated GNSs within the 400–900 nm spectral range. j) Peptide density per surface area of GzmB‐activated nanosensors fabricated at different peptide‐to‐GNS ratios in conjugation (n = 3). k) Time‐dependent changes in extinction ratio (700 nm/520 nm) of GzmB‐activated plasmonic nanosensors with different peptide densities in the presence of 50 nm GzmB (n = 3). l) Corresponding extinction ratio of the nanosensors measured 1 h after coincubation with 50 nm GzmB. m) Extinction ratio (700 nm/520 nm) of GzmB‐activated plasmonic nanosensors incubated at different GzmB concentrations. n) Time‐dependent changes in extinction ratio (700 nm/520 nm) of GzmB‐activated plasmonic nanosensors in the presence of different proteases or BSA (n = 3). Data are presented as the mean ± standard deviation. The statistical analysis for Figure 1c,d was conducted using a two‐tailed Student's t‐test. The statistical analysis for Figure 1j–l was conducted using a one‐way ANOVA with Tukey post‐hoc tests. The statistically significant difference is represented as the asterisk (ns: non‐significant, ^**^: *p* < 0.01, ^***^: *p* < 0.001, ^****^: *p* < 0.0001).

Tuning interparticle proximity of GNSs to induce plasmon coupling and thus modulate optical responses in the NIR wavelength range^[^ ^43^, ^52^, ^53^, ^54^, ^55^, ^56^ ^]^ upon exposure to GzmB can be potentially harnessed for designing plasmonic nanosensors in molecular PA imaging of T cell activity. To exploit this, we synthesized GzmB‐activated plasmonic nanosensors consisting of a GNS core functionalized with peptide sequences that contain the GzmB substrate (Iso‐Glu‐Phe‐Asp, IEFD). Upon enzymatic cleavage by GzmB, these nanosensors undergo aggregation via destabilization, facilitating plasmonic coupling between proximal nanosensors and resulting in enhanced optical responses at NIR wavelengths.^[^ ^54^, ^56^, ^57^, ^58^ ^]^ Specifically, we fabricated 15 nm‐sized GNSs using the Turkevich method^[^ ^59^, ^60^ ^]^ and conjugated the peptide sequences to the GNS surface via gold–thiol bonds at varying peptide‐to‐GNS stoichiometric ratios in the conjugation process (**Figure** **1**a; Figure S1, Supporting Information). Notably, particle aggregation was observed when the peptide‐to‐GNS ratio was below 509, indicating insufficient surface coverage of peptides to maintain colloidal stability (Figure S1, Supporting Information). When functionalizing GNSs with GzmB cleavable peptides at a peptide‐to‐GNS ratio of 763, GNSs exhibited a slight increase of hydrodynamic diameter due to the presence of the peptides on the surface of GNSs, while exhibiting a comparable optical signature with unconjugated GNSs (Figure 1b,c).

We next assessed if GzmB cleavable peptide‐conjugated GNSs (GzmB‐activated plasmonic nanosensors) can undergo an aggregation behavior and subsequently change their optical responses in the NIR wavelength range upon exposure to GzmB. In the presence of GzmB, the diameter of the GNSs increased via aggregation, consequently enhancing NIR light responses (Figure 1d–f). To understand the mechanism of the aggregation‐mediated amplification of optical activity of nanosensors within the NIR spectral range, we performed finite‐difference time‐domain (FDTD) simulations on a randomly aggregated GNS with an overall dimension of ≈100 nm. The simulated aggregate exhibited strong localized electric field enhancement at 700 nm, attributed to plasmon coupling between proximate particles (Figure 1g). This plasmonic interaction led to dominant optical absorption at NIR wavelengths with minimal optical scattering (Figure 1h,i). The enhanced absorption efficiency arising from aggregation implies that GzmB‐mediated activation of nanosensors enables efficient conversion of incident laser pulses into PA signals. Although this simplified model does not fully capture the heterogeneous aggregation morphologies and size distributions observed experimentally, it effectively illustrates that plasmon coupling between adjacent GNSs leads to strong localized electric field enhancement and subsequently enhanced NIR light absorption.

Additionally, we investigated whether the peptide density, i.e., peptide valency, modulates the proteolysis‐mediated aggregation kinetics of the nanosensors. Specifically, we varied the peptide‐to‐GNS ratio from 763 to 2290 in conjugation, yielding a surface density per GNS surface area from 0.43 to 0.72 nm^−2^ (Figure 1j; Figure S2, Supporting Information). Given that GzmB‐mediated nanosensor aggregation enhances optical responses at NIR wavelengths, we investigated how varying peptide densities influence this aggregation by performing ratiometric analyses in their optical signature of the nanosensors. In the absence of GzmB, nanosensors exhibited a dominant extinction peak at ≈520 nm, whereas upon exposure to GzmB, nanosensor aggregation occurred, resulting in the emergence of a shoulder extinction peak near 700 nm (Figure 1f). We thus quantified the extinction ratio of 700 to 520 nm to evaluate the extent of interpaticle plasmon coupling induced by GzmB‐mediated nanosensor aggregation, as the absorption of ≈700 nm lies within the NIR region optimal for high‐contrast PA imaging. The results showed that nanosensors with lower peptide density exhibited more robust aggregation, as indicated by a higher extinction ratio (Figure 1k,l). This suggests that reduced peptide density enhances the accessibility of GzmB to its substrate on the nanosensor surface by minimizing steric hindrance. In the absence of GzmB, all nanosensor variants remained stable with no aggregation, as indicated by comparable extinction ratio values (Figure S3, Supporting Information). The nanosensors with lower peptide density (≈0.43 nm^−2^) detected were able to detect GzmB as low as 1.56 nm (Figure 1m; Figure S4, Supporting Information). Furthermore, as shown in Figure 1n, the nanosensors demonstrated high specificity for GzmB, showing minimal responsiveness in the presence of denatured GzmB, tumor‐associated proteases such as matrix metalloproteinase‐7 (MMP‐7) and MMP‐9, and cell apoptosis‐related protease caspase‐3 (Cas‐3). Additionally, the presence of bovine serum albumin (BSA) did not induce nanosensor aggregation. Together, these results suggest that our nanosensor platform could be utilized for the detection of GzmB proteolytic activity via PA imaging, leveraging aggregation‐induced amplification of optical absorption in the NIR window.

### 2.2 Enhanced Optical Activity Resulting from Aggregation of GzmB‐Activated Plasmonic Nanosensors Leads to Amplified PA Responses in the NIR Wavelength Range

**Figure 2:**
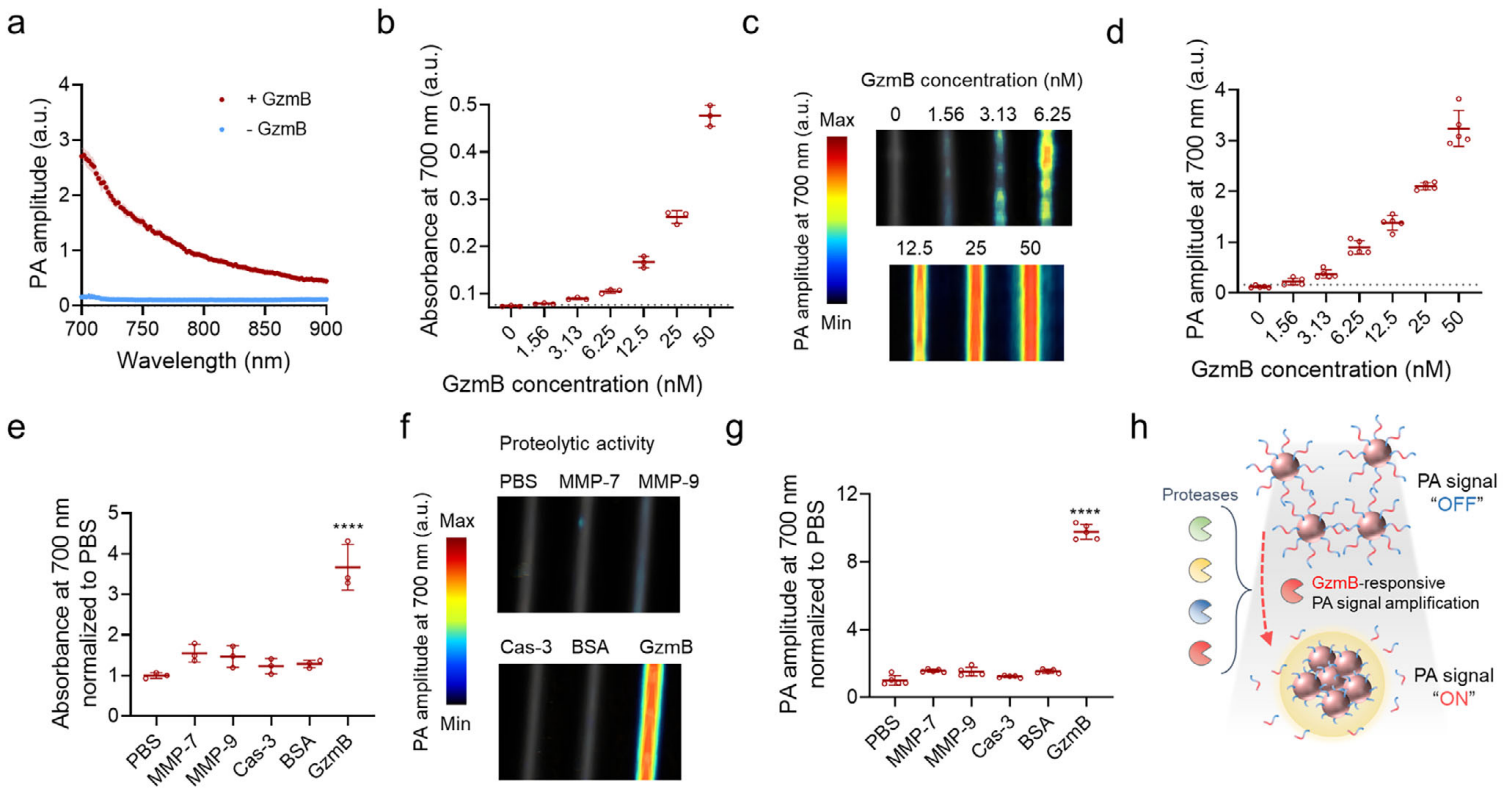
Enhanced optical activity resulting from aggregation of GzmB‐activated plasmonic nanosensors leads to amplified PA responses in the NIR wavelength range. a) PA signals from GzmB‐activated plasmonic nanosensors within the 700–900 nm spectral range in the presence/absence of GzmB (n = 4). b–d) Absorbance (b, n = 3) and PA responses (c, d, n = 5) from GzmB‐activated plasmonic nanosensors at a wavelength of 700 nm were measured 1 h after incubation with different concentrations of GzmB. e–g) Absorbance (e, n = 3) and PA responses (f, g, n = 5) from GzmB‐activated plasmonic nanosensors at a wavelength of 700 nm were measured 1 h after incubation with different proteases or BSA. h) Schematic illustration of peptide cleavage‐mediated PA signal activation of GzmB‐activated plasmonic nanosensors, highlighting significant amplification by GzmB among diverse proteases. Data are presented as the mean ± standard deviation. The statistical analysis for Figure 2e–g was conducted using a one‐way ANOVA with Tukey post‐hoc tests. The statistically significant difference is represented as the asterisk (^****^: *p* < 0.0001).

As GzmB‐activated plasmonic nanosensors can enhance optical absorption within the NIR spectral range upon exposure to GzmB, we went on to demonstrate whether the amplified optical response from the nanosensor could impart PA responses within the NIR window. To reflect the practical wavelength range of PA imaging, the nanosensors were characterized in 700–900 nm wavelength range. Upon peptide cleavage by GzmB, the nanosensors exhibited significantly higher PA amplitudes within this spectral range, compared to untreated, monodispersed nanosensors (**Figure** **2**a). The amplification of optical and subsequent generation of PA responses by GzmB‐activated nanosensors was dependent on the concentration of GzmB, enabling detection of GzmB at levels as low as 3.13 nm in PA imaging (Figure 2b–d). The slightly higher detection limit observed in PA measurements, relative to optical measurements (1.56 nm), could result from variations in acoustic coupling efficiency, detector sensitivity, and pulse‐to‐pulse laser fluence fluctuations inherent to PA imaging.

Next, we evaluated whether the GzmB‐activated plasmonic nanosensors could generate amplified PA responses with specificity for GzmB, distinguishing it from MMP‐7, MMP‐9, and Cas‐3. In the presence of GzmB, the nanosensors exhibited significantly enhanced optical activity and amplified PA signals and contrast at 700 nm, while showing minimal responses to other proteases and BSA (Figure 2e–g). These results collectively indicate that GzmB‐activated nanosensors could selectively report the proteolytic activity of GzmB as a PA signal output at NIR wavelengths through aggregation‐mediated amplification of optical responses (Figure 2h).

### 2.3 GzmB‐Activated Plasmonic Nanosensors Report Both Activation and Antitumor Responses of Cytotoxic T Cells by Producing Enhanced Optical and PA Signals in the NIR Wavelength Range

**Figure 3:**
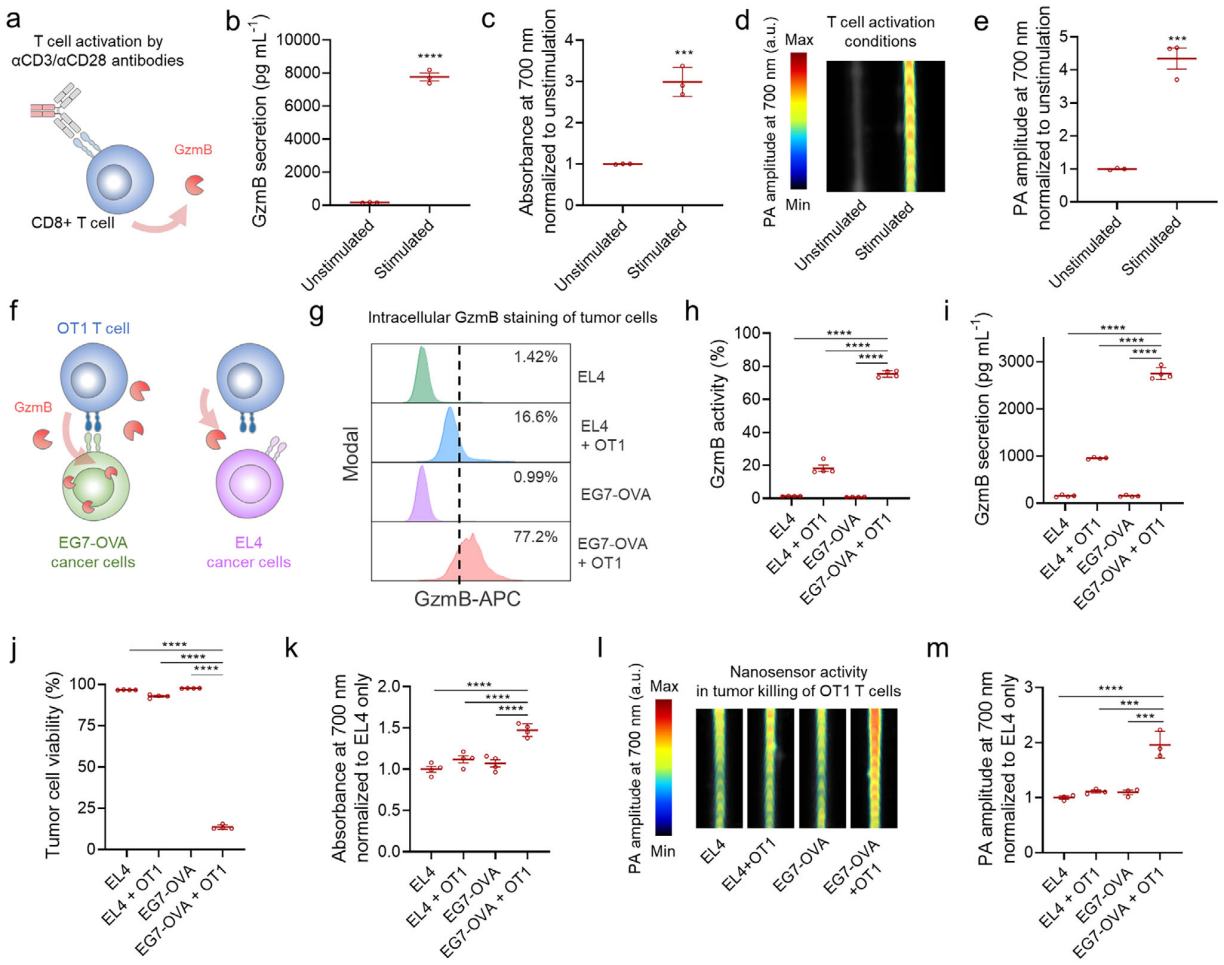
GzmB‐activated plasmonic nanosensors report both activation and antitumor activity of cytotoxic T cells by producing enhanced optical and PA signals in the NIR wavelength range. a) Schematic illustration of GzmB secretion from CD8^+^ T cells upon stimulation with αCD3/αCD28 antibodies. b) GzmB concentration in media from CD8^+^ T cells with/without CD3/CD28 stimulation (n = 3). c–e) Absorbance (c, n = 3) and PA responses (d, e, n = 3) of GzmB‐activated plasmonic nanosensors at 700 nm when treated with the media for 1 h (n = 3). f) Schematic illustration of the co‐culture killing assay using TCR‐transgenic OT1 T cells and tumor cells either lacking (EL4) or expressing (EG7‐OVA) the OVA antigen. g,h) Intracellular GzmB expression levels of EL4 or EG7‐OVA tumor cells following co‐incubation with/without OT1 T cells (n = 4). i) GzmB concentration in the co‐culture media (n = 4). j) Viability of EL4 or EG7‐OVA tumor cells after coincubation with/without OT1 T cells (n = 4). k‐m) Absorbance (k, n = 4) and PA responses (l, m, n = 3) from GzmB‐activated plasmonic nanosensors at 700 nm after 1‐h incubation with the co‐culture media. Data are presented as the mean ± standard deviation. The statistical analysis for Figure 3b–e was conducted using a two‐tailed Student's t‐test. The statistical analysis for Figure 3h–k and m was conducted using a one‐way ANOVA with Tukey post‐hoc tests. The statistically significant difference is represented as the asterisk (ns: non‐significant, ^***^: *p* < 0.001, ^****^: *p* < 0.0001).

Upon recognizing cells that express a cognate antigen via T cell receptor (TCR) engagement, cytotoxic CD8⁺ T cells become activated, resulting in the formation of an immunological synapse and the directed secretion of cytotoxic effector molecules, GzmB, toward target cells.^[^ ^5^ ^]^ This release of GzmB subsequently induces apoptosis in target cells, such as tumor cells.^[^ ^5^ ^]^ To determine whether GzmB‐activated plasmonic nanosensors could report physiologically relevant GzmB activity, we evaluated their response to GzmB secreted by mouse cytotoxic T cells. Media were collected from CD8⁺ T cells that were either unstimulated or stimulated with CD3/CD28, confirming the GzmB secretion only in the stimulated group (**Figure** **3**a,b). When incubated with conditioned media, the nanosensors exhibited significant time‐dependent amplification of optical and PA signals at NIR wavelengths in response to GzmB from activated cytotoxic T cells, compared to minimal responses in media from unstimulated cells (Figure 3c–e; Figures S5–S7, Supporting Information).

Next, we investigated whether the nanosensors could report the antitumor cytotoxic activity of CD8⁺ T cells by elevating optical and PA responses in the NIR window. To validate this, we co‐incubated ovalbumin (OVA)‐specific OT1 CD8⁺ T cells with either cognate antigen‐expressing tumor cells (EG7‐OVA) or antigen‐lacking tumor cells (EL4) overnight. Flow cytometry confirmed that EG7‐OVA cells exhibited significantly higher intracellular GzmB levels when co‐cultured with OT1 T cells, compared to EL4 tumor cells with OT1 T cells or tumor cells alone (Figure 3f–h). Furthermore, as validated by ELISA, substantial GzmB secretion was observed when OT1 T cells were co‐cultured with EG7‐OVA tumor cells, compared to co‐cultures with EL4 tumor cells or tumor cells alone (Figure 3i). This antigen‐dependent GzmB release from OT1 T cells led to specific cytotoxicity against EG7‐OVA tumor cells as characterized by flow cytometry (Figure 3j). To assess the capability of GzmB‐activated plasmonic nanosensors in monitoring the antitumor activity of OT1 T cells against target tumor cells, the nanosensors were incubated with conditioned media collected from OT1 T cells co‐incubated with either EG7‐OVA or EL4 tumor cells, as well as from tumor cells cultured alone. As shown in Figure 3k; Figure S8 (Supporting Information), the nanosensors significantly amplified optical absorption at 700 nm in media from OT1 T cells co‐cultured with EG7‐OVA cells compared to the other groups. Correspondingly, this enhanced optical response translated into stronger PA signals and contrast at NIR wavelengths in the media from OT1 T cells co‐cultured with EG7‐OVA tumor cells relative to other conditions (Figure 3m; Figure S9, Supporting Information). The smaller PA signal change (Figure 3m) versus tumor GzmB expression (Figure 3h) likely reflects that only secreted, catalytically active GzmB activates the nanosensors. Taken together, these results demonstrate that our nanosensors effectively detect the antitumor activity of cytotoxic T cells by generating amplified optical and PA signals within the NIR window.

### 2.4 GzmB‐Activated Plasmonic Nanosensors Detect Therapeutic Antitumor Responses Following Adoptive T Cell Transfer via US/PA Imaging, Preceding Observable Changes in Tumor Burden

As GzmB‐activated plasmonic nanosensors can specifically detect tumor killing of cytotoxic T cells by producing PA signals and contrast in vitro, we thus went on to demonstrate whether the nanosensors could noninvasively report antitumor T cell activities in preclinical murine tumor models following ACT via US/PA imaging in vivo. As an initial assessment, systemic administration of nanosensors resulted in no significant changes in body weight over 15 days compared to saline‐treated controls, indicating biocompatibility (Figure S10, Supporting Information). We then established subcutaneous EG7‐OVA and EL4 tumors in mice and monitored their PA responses and tumor volume changes before and after intravenous nanosensor injection (Figure S11, Supporting Information). In the absence of ACT, both tumor types showed comparable PA signals at 700 nm before and after nanosensor administration, with no significant differences in tumor growth, suggesting that nanosensors are not actuated and therefore do not produce detectable PA signal differences between OVA‐positive and OVA‐negative tumors (Figure S11, Supporting Information).

**Figure 4:**
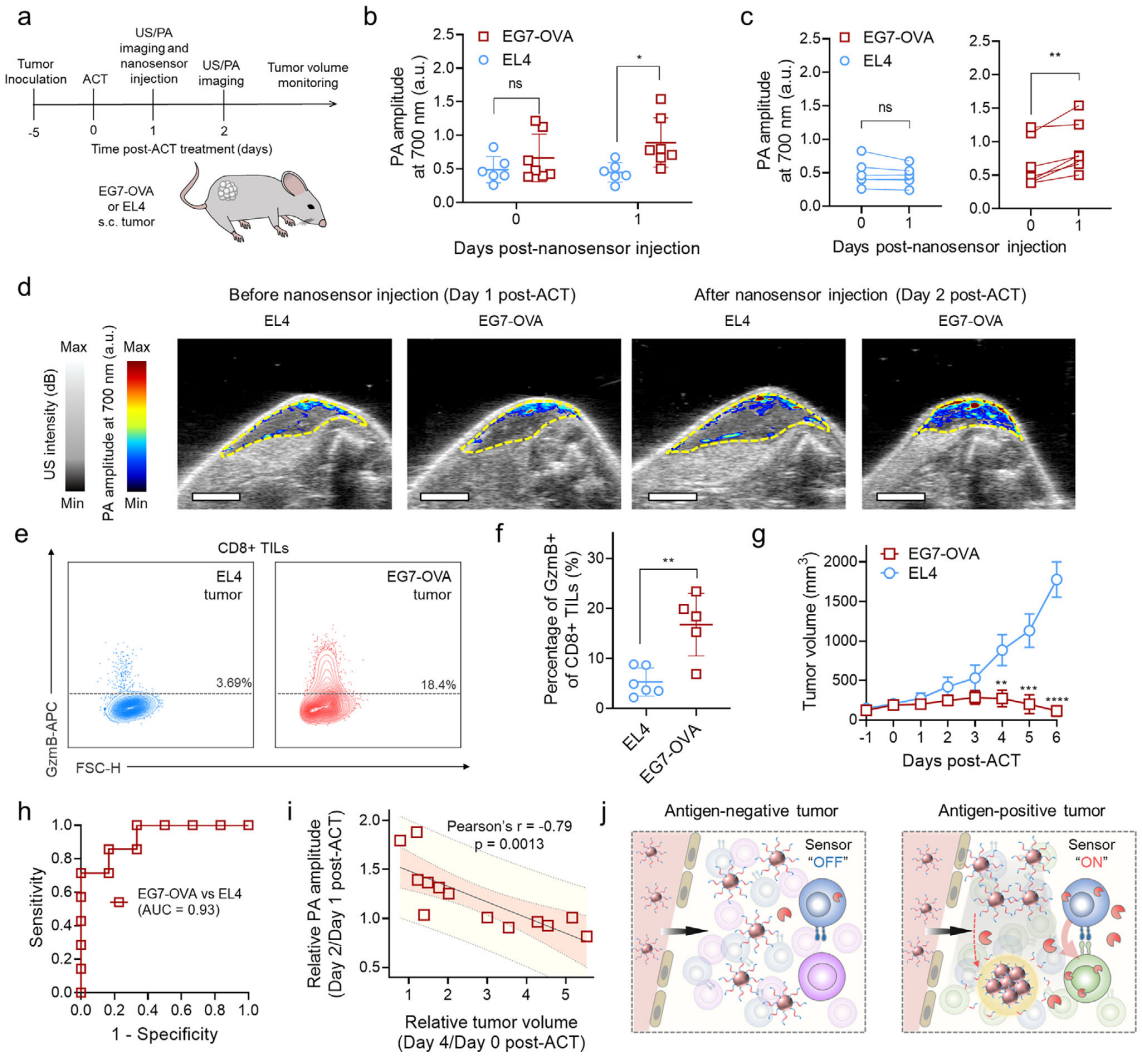
GzmB‐activated plasmonic nanosensors detect therapeutic antitumor responses following adoptive T cell transfer via US/PA imaging, preceding observable changes in tumor burden. a) Schematic illustration of the workflow of ACT and US/PA imaging with GzmB‐activated nanosensor administration. b–d) PA responses (c), PA signal comparison, and US/PA images (d) of EL4 or EG7‐OVA tumors in mice treated with ACT before and after systemic nanosensor injection (n = 6 or 7, respectively). Scale bars are 4 mm. e,f) Representative flow plots (e) and percentages (f) of live GzmB^+^CD8^+^ T cells isolated from EL4 or EG7‐OVA tumors at day 2 post‐ACT (n = 6 or 5, respectively). g) Measured volume changes of the EL4 or EG7‐OVA tumors (n = 6 or 7, respectively). h) A receiver‐operator characteristic (ROC) curve showing the diagnostic specificity and sensitivity of the nanosensors in differentiating between responders and non‐responders upon ACT from PA signals at day 2 post‐ACT. i) Correlation analysis between relative tumor volume (day 4/day 0 post‐ACT) and relative PA amplitude (day 2/day 1 post‐ACT, i.e., before and after intravenous nanosensor injection). The linear regression model was fitted to the data points from individual mice (n = 13) with 95% confidence and 95% prediction bands. j) Schematic illustration of nanosensor‐mediated detection of antitumor activity of T cells via US/PA imaging of antigen‐negative or antigen‐positive tumors following ACT. Data are presented as the mean ± standard deviation. The statistical analysis for Figure 4b–f was conducted using an unpaired two‐tailed Student's t‐test. The statistical analysis for Figure 4c was conducted using a paired two‐tailed Student's t‐test. The statistical analysis for Figure 4g was conducted using a two‐way ANOVA with Sidak's post‐test. The statistically significant difference is represented as the asterisk (ns: non‐significant, ^*^: *p* < 0.05, ^**^: *p* < 0.01, ^***^: *p* < 0.001, ^****^: *p* < 0.0001).

Next, to evaluate whether the nanosensors can report antitumor activity of CD8⁺ tumor‐infiltrating lymphocytes (TILs), mice bearing EG7‐OVA or EL4 tumors received ACT with OVA‐specific OT1 T cells, followed by intravenous nanosensor injection on day 1 post‐ACT (**Figure** **4**a). Although both tumors displayed similar PA signals prior to nanosensor injection, EG7‐OVA tumors exhibited significantly elevated PA signals and contrast at 700 nm one day after nanosensor injection (day 2 post‐ACT), compared to EL4 tumors, suggesting enhanced antitumor activity of CD8⁺ TILs in EG7‐OVA tumors (Figure 4b–d). PA signals were predominantly localized at the tumor periphery, likely due to restricted nanosensor delivery to the tumor core, influenced by higher vascular density at the periphery and elevated interstitial fluid pressure in the core,^[^ ^61^, ^62^ ^]^ as well as laser light attenuation with depth. Notably, the rise in PA amplitude within the responder group exhibited intragroup variation, reflecting differences in T cell infiltration, nanosensor delivery, and tumor microenvironmental properties. Flow cytometry analysis confirmed that EG7‐OVA tumors exhibited higher GzmB expression in CD8⁺ TILs on day 2 post‐ACT compared to EL4 tumors, supporting the increased PA signals from the antigen‐positive tumors (Figure 4e,f). Additionally, a higher infiltration of OT1 T cells into EG7‐OVA tumors compared to EL4 tumors was validated by flow cytometry using CD45.1 congenic mice (Figure S12, Supporting Information). Tumor volume measurements following ACT showed a significant difference on day 4 post‐ACT, with tumor regression occurring only in antigen‐positive tumors, EG7‐OVA (Figure 4g; Figure S13, Supporting Information). Notably, the nanosensors detected therapeutic responses prior to detectable changes in tumor burden. Receiver operator characteristic (ROC) curve analysis of the nanosensor signals from tumors at day 2 post‐ACT yielded an area under the curve (AUC) of 0.93, indicating that the nanosensors can distinguish antitumor TIL activity with high sensitivity and specificity (Figure 4h). Furthermore, increases in PA signal amplitudes from ACT‐treated tumors following nanosensor administration were significantly anticorrelated with tumor volume changes measured between day 0 to day 4 post‐ACT (Pearson's coefficient = – 0.79, p = 0.0013), a period during which distinct differences in tumor growth emerged between EL4 and EG7‐OVA tumors (Figure 4i). Given the ability of the nanosensors to differentiate treatment responders from non‐responders through early detection of antitumor T cell activity and to correlate nanosensor signals with subsequent tumor regression (Figure 4h,i), these findings collectively demonstrate that GzmB‐activated plasmonic nanosensors can noninvasively detect the protease activity of intratumoral T cells and serve as predictive tools for assessing therapeutic efficacy in ACT.

## 3 Conclusion 

In this study, we designed and validated GzmB‐activated plasmonic nanosensors as noninvasive reporters of cytotoxic T cell activity through US/PA imaging. Upon exposure to GzmB, the nanosensors undergo peptide cleavage‐mediated aggregation, leading to enhanced plasmonic coupling and amplified optical absorption within the NIR window. This aggregation‐induced enhancement of optical activity of the nanosensors led to enhanced PA signals, enabling sensitive and specific detection of antitumor activities of T cells in both in vitro and in vivo settings. By tuning peptide surface density, we demonstrated that nanosensor aggregation and thereby signal amplification can be regulated to enhance detection sensitivity, while ensuring high specificity for GzmB over other tumor‐associated or cell apoptosis‐related proteases. Moreover, the nanosensors selectively responded to physiologically secreted GzmB from activated cytotoxic T cells and detected GzmB released by antigen‐specific T cells upon recognition of target antigen‐expressing tumor cells, correlating with increased PA signals and T cell cytotoxicity. In a murine tumor model, systemic administration of GzmB‐activated nanosensors enabled early, noninvasive monitoring of therapeutic responses following ACT, prior to observable tumor regression. Tumors expressing the target antigen exhibited enhanced PA signals due to elevated GzmB+CD8⁺ T cell infiltration, confirming the nanosensor's capacity to monitor functional T cell activity in situ. Together, these findings establish GzmB‐activated plasmonic nanosensors as a platform for real‐time assessment of antitumor immune responses.

This sensor platform offers potential for enhancing the precision and monitoring of immunotherapies. For example, the nanosensors can be utilized in immune checkpoint blockade therapy by enabling early and spatially resolved detection of cytotoxic T cell‐mediated tumor killing activity. Furthermore, future studies will extend US/PA imaging to later stages to evaluate longitudinal nanosensor performance and precisely monitor progressive T cell activity in tumors, while correlating these longitudinal nanosensor‐derived signals with tumor regression or relapse. Moreover, the modular design of this platform, enabled by the tunable nature of peptide sequences, allows the platform to be adapted for detecting a variety of disease‐associated proteases derived from either mouse or human (Figure S14, Supporting Information), thereby expanding its practical utility across diverse immunological and pathological conditions. However, a potential limitation of this nanosensor is that in vivo variability in nanosensor aggregation could unpredictably alter the spectrum of PA signal, which may affect spectral unmixing. Addressing this limitation in future designs could further enhance the versatility and quantitative accuracy of this platform.

## Conflict of Interest

G.A.K. reports equity or consulting roles for Sunbird Bio, Port Therapeutics, Send Biotherapeutics, and Ridge Biotechnologies. This study could affect his personal financial status. The terms of this arrangement have been reviewed and approved by Georgia Tech in accordance with its conflict‐of‐interest policies.

## Author Contributions

M.K. and S.Y.E. conceived the idea. S.Y.E. supervised all aspects of the project. M.K., A.Z., G.A.K., and S.Y.E. designed the experiments. M.K., S.S., A.Z., P.S.P., S.S., M.C., S.F., S.F., and M.B. performed all experiments. M.K., S.S., A.Z., J.K., G.A.K., and S.Y.E. analyzed the data. M.K. and S.Y.E. wrote the draft of the manuscript. All authors discussed and commented on the manuscript.

## Ethics Statement

All animal experiments in this study were approved by the Institutional Animal Care and Use Committee (IACUC) at the Georgia Institute of Technology (protocol number: A100281).

## Supporting information



Supporting Information

## Data Availability

The data that support the findings of this study are available from the corresponding author upon reasonable request.;
