# Supplementary material for: Protease‐Activated Plasmonic Nanosensors for Predictive Ultrasound‐Guided Photoacoustic Imaging of Tumor Responses to Adoptive T Cell Therapy
Source: Adv Sci (Weinh). 2025 Dec 14;13(11):e15111. doi: 10.1002/advs.202515111 (PMC12931215; doi:10.1002/advs.202515111)
Supplement: Supplementary file 1 — Supporting Information [file ADVS-13-e15111-s001.docx]

**Supporting Information**

**Protease-Activated Plasmonic Nanosensors for Predictive Ultrasound-Guided Photoacoustic Imaging of Tumor Responses to Adoptive T Cell Therapy**

*Myeongsoo Kim^1,2^, Seoyoon Song^1^, Ali Zamat^1^, Paul S. Pelkowski^1^, Shivashankar Subramanian^1^, Melissa Cadena^1^, Sydney Fabrega^1^, Meredith Brienen^1^, Jinhwan Kim.^3,4^, Gabriel A. Kwong^1,2,5,6,7,8^, Stanislav Y. Emelianov^1,2,5,6,8,9,✉^*

*^1^Wallace H. Coulter Department of Biomedical Engineering, Georgia Institute of Technology and Emory University School of Medicine, Atlanta, GA, USA*

*^2^Petit Institute for Bioengineering and Bioscience, Georgia Institute of Technology, Atlanta, GA, 30332, US*

*^3^Department of Biomedical Engineering, University of California Davis, Davis, CA, USA*

*^4^Department of Surgery, School of Medicine, University of California Davis, Sacramento, CA, USA*

*^5^Institute for Electronics and Nanotechnology, Georgia Institute of Technology, Atlanta, GA, USA*

*^6^Integrated Cancer Research Center, Georgia Institute of Technology,*

*^7^Georgia Immunoengineering Consortium, Emory University and Georgia Institute of Technology, Atlanta, GA, USA.*

*^8^Winship Cancer Institute, Emory University, Atlanta, GA, USA*

*^9^School of Electrical and Computer Engineering, Georgia Institute of Technology, Atlanta, GA, USA*

^✉^ Corresponding Author: Stanislav. Y. Emelianov ([stas@gatech.edu](mailto:stas@gatech.edu))

**Supplementary Note 1.** Experimental Details

**1.1. Synthesis of 15-nm-sized plasmonic gold nanospheres**

Plasmonic gold nanospheres (15 nm) were synthesized via Turkevich method^[1,2]^. Specifically, 150 mL of 1 mM gold (III) chloride trihydrate (Sigma Aldrich) aqueous solution was boiled under magnetic stirring. To this solution, 7.5 mL of 77.6 mM sodium citrate (Sigma Aldrich) aqueous solution was rapidly injected, followed by magnetic stirring for 10 minutes and cooling down to room temperature. The concentration of the nanospheres was adjusted to 8.17 nM before use.

**1.2. Fabrication of granzyme B (GzmB)-activated plasmonic nanosensors**

To fabricate GzmB-activated plasmonic nanosensors, GzmB-cleavable peptides (AIEFDSGC, GenScript) were conjugated to the surface of gold nanospheres. Specifically, the peptides in dimethyl sulfoxide (DMSO, Sigma Aldrich) were mixed with 20 mL of gold nanospheres at varying peptide-to-gold nanosphere stoichiometric ratio ranging from 127 to 2,290, followed by shaking for 30 minutes. The peptide-conjugated gold nanospheres (GzmB-activated plasmonic nanosensors) were washed via centrifugation (11,000 rpm for 30 minutes) and then dispersed in 2 mL of phosphate buffered saline (Corning, PBS) solution. The hydrodynamic diameter of GzmB-activated plasmonic nanosensors with varying peptide densities was characterized via dynamic light scattering (Zetasizer Nano ZS, Malvern Instruments Ltd.).

To quantify the peptide density per gold nanosphere, the amine group at N-terminus was conjugated with Cy5 as a fluorescent reporter. Specifically, 1 mL of nanosensors was washed via ultracentrifugation with a membrane for 50 kDa cutoff (Amicon® Ultra-15 Centrifugal Filter Unit, Ultracel-50 Membrane) to remove unbound peptides. The nanosensors were then redispersed in 1 mL of PBS. Next, 100 μL of the nanosensor in PBS was mixed with 900 μL of DMSO. To this solution, 10 μL of 0.2 mM Cy5-NHS ester (BroadPharm) in DMSO was added, followed by shaking in dark for 1 hour. The Cy5-conjugated nanosensors were washed with DMSO twice via centrifugation to remove unbound Cy5 dyes, followed by suspension in 1 mL of DMSO. By comparing the intensity of peak absorption of Cy5 and gold nanosphere in Cy5-conjugated nanosensors via UV-vis-NIR spectroscopy (Evolution 220, Thermo Scientific), the peptide density per gold nanosphere in nanosensor was quantified.

**1.3. Characterization for enhanced optical activities in the NIR wavelength range from aggregation of GzmB-activated plasmonic nanosensors upon exposure to GzmB**

To assess optical activity of the nanosensors with varying peptide densities upon exposure to GzmB (Recombinant murine GzmB, ReproTech), the nanosensors were redispersed in PBS, adjusting the concentration of approximately 14 nM. Next, the nanosensors in 60 µL with different peptide densities were mixed with 30 µL of 150 nM GzmB in PBS. The extinction ratio of 700 nm to 520 nm as an indicator of plasmon coupling intensity resulting from GzmB-mediated nanosensor aggregation was measured every minute via a well plate reader (Synergy HY, BioTek). Next, to characterize GzmB concentration-dependent nanosensor aggregation, 60 µL of GzmB-activated nanosensors with a peptide density per surface area of gold nanosphere of 0.43 nm^-2^ was added to 30 µL of GzmB with different concentrations in PBS or PBS control. The extinction ratio of 700 nm to 520 nm was measured every minute at 37 ^o^C via a well plate reader (Synergy HY, BioTek). The nanosensor specificity for GzmB was assessed by characterizing time dependent changes in extinction ratio of 700 nm to 520 nm of the nanosensors at 37 ^o^C in the presence of 50 nM GzmB, 70 nM caspase-3 (recombinant caspase-3 protein, Abcam), 50 nM matrix metalloproteinase (MMP)-7 (recombinant human MMP-7, BioLegend), 50 nM MMP-9 (recombinant human MMP-9, BioVision), 50 nM denatured GzmB (heated at 80 ^o^C for 20 minutes), 1% bovine serum albumin (Sigma Aldrich), or PBS control.

**1.4. Numerical simulation**

Optical cross-sections, including extinction, absorption, and scattering for aggregated gold nanospheres were calculated by a FDTD simulation (Lumerical Inc.). For the FDTD computational analysis, the surrounding temperature was 300 K. The surrounding medium was chosen as water. The value in Johnson and Christy^[3]^ was used for dielectric constant and refractive index of gold. A total-field/scattered-field (TF/SF) source with a 400-900 nm wavelength range was utilized to calculate their optical extinction, absorption, and scattering cross-sections. Randomly aggregated gold nanospheres with a dimension of approximately 100 nm were used as a simulation model. The optical extinction, absorption, and scattering cross-sections for the nanosphere aggregate were calculated with an average value from the optical cross-sections under light excitation at s- or p-polarization, while setting a maximum mesh step as 0.5 nm.

**1.5. Characterization for enhanced PA responses within the NIR window from aggregation of plasmonic nanosensors with specificity for GzmB**

To assess enhanced PA responses from amplified optical signals from the nanosensors upon exposure to GzmB, Vevo2100/LAZR imaging system (FujiFilm VisualSonics Inc.) with a LZ250 US transducer integrated with optical-fiber light delivery system was employed. To characterize GzmB concentration-dependent PA signal amplification of the nanosensors, 60 µL of ~14 nM nanosensor with a peptide density per surface area of gold nanosphere of 0.43 nm^-2^ was added to 30 µL of GzmB with different concentrations in PBS or PBS control, followed by incubation for 1 hour at 37 ^o^C. Next, the absorbance at 700 nm of the treated nanosensors was measured via a well plate reader (Synergy HY, BioTek). The nanosensors were then added to polyethylene tubes (BD Intramedic) in a 3D-printed scaffold. The scaffold with the tubes was placed in the Vevo2100/LAZR imaging system and PA amplitudes of the nanosensors at 700 nm were quantified. Laser pulses were generated by a Q-switched Nd:YAG-pumped optical parametric oscillator (OPO) laser (pulse duration: 7 ns, frame rate: 20 Hz). The gain in PA images and B-modes was set to 40 dB and 18 dB, respectively. To assess the specificity of the nanosensor for GzmB, the nanosensor was incubated for 1 hour at 37 ^o^C in the presence of 50 nM GzmB, 70 nM caspase-3 (recombinant caspase-3 protein, Abcam), 50 nM matrix metalloproteinase (MMP)-7 (recombinant human MMP-7, BioLegend), 50 nM MMP-9 (recombinant human MMP-9, BioVision), 1% bovine serum albumin (Sigma Aldrich), or PBS control. Absorbance at 700 nm was measured, and corresponding PA responses were acquired using the same imaging parameters.

**1.6. Cell culture**

EL4 or EG7-OVA cells were cultured in RPMI 1640 (Cytiva) supplemented with 10% fetal bovine serum (FBS, Sigma Aldrich), 1% penicillin-streptomycin (PS, Sigma Aldrich), 0.05 mM 2-mercaptoehanol (Sigma Aldrich), 10 mM HEPES (Thermo Fisher Sicentific), and 0.4 mg mL^-1^ G418 (only for EG7-OVA, Thermo Fisher Sicentific). Murine CD8+ T cells or OT1 T cells were isolated (CD8a T cell isolation kit, Milltenyi Biotech, Inc.) from spleens of female C57BL/6 mice (5 to 10 weeks, The Jackson Laboratory) or female OT1 transgenic mice (C57BL/6-Tg(TcraTcrb)1100Mjb/J, 5 to 16 weeks, The Jackson Laboratory). Isolated T cells were activated for two days using anti-CD3e and anti-CD28 antibodies (BD Biosciences, clone 145-2C11 and 37.51, respectively). For OT1 T cells, the cells were cultured for up to 5 days at 1.2 to 1.5 million cells per mL, using murine T cell media, RPMI 1640 supplemented with 10% FBS, 1% PS, 1% non-essential amino acids (Thermo Fisher Sicentific), 1% sodium pyruvate (Thermo Fisher Sicentific), 0.05 mM 2-mercaptoethanol (Sigma Aldrich), 100 units per mL IL-2 (ReproTech or Frederick National Laboratory), and 1 µg mL^-1^ ovalbumin (OVA-257-264, chicken, Sigma Aldrich, only supplemented on the day of isolation).

**1.7. Characterization for GzmB-activated plasmonic nanosensors in vitro**

To assess the capability of GzmB-activated plasmonic nanosensors to report T cell activation, media were collected from murine CD8⁺ T cells cultured with or without CD3/CD28 stimulation. The collected media were used for either GzmB quantification via ELISA (ThermoFisher Scientific) or for evaluating nanosensor responsiveness. For the latter, conditioned media were added to the nanosensor solution, after which absorbance at 700 nm was measured at one-minute intervals. Additionally, by adding the media treated with nanosensors to polyethylene tubes in a 3D-printed scaffold via Vevo2100/LAZR imaging system (FujiFilm VisualSonics Inc.) with an LZ250 US transducer (center frequency: 21 MHz, bandwidth range: 13-24 MHz, focal depth: 10 mm) integrated with optical-fiber light delivery system. Laser pulses were generated by a Q-switched Nd:YAG-pumped optical parametric oscillator (OPO) laser (laser fluence: ~10 mJ cm^-2^, pulse duration: 7 ns, pulse repetition frequency: 20 Hz). The gain in PA images and B-modes was set to 40 dB and 18 dB, respectively.

To characterize the ability of the nanosensors to detect antitumor responses of cytotoxic T cells, EL4 or EG7-OVA tumor cells were washed with T cell media twice, and 150,000 cells were added to a 24-well plate. Next, OT1 T cells were washed with T cell media, and 1,500,000 cells were added to the tumor cells (effector-to-target ratio = 10:1). After overnight incubation, media was collected via centrifugation. Conditioned media was used for either GzmB ELISA or for evaluating nanosensor activity in optical and PA responses as described above.

**1.8. Characterization for intracellular GzmB expression of tumor cells and T cell cytotoxicity**

To evaluate cytotoxicity of OT1 T cells against EL4 or EG7-OVA tumor cells, EL4 or EG7-OVA tumor cells were stained with CFSE (Thermo Fisher Scientific) according to the manufacturer’s instructions. Next, 150,000 CFSE-stained tumor cells were added to a 24-well plate. Next, OT1 T cells were washed with T cell media, and 1,500,000 cells were added to the tumor cells (effector-to-target ratio = 10:1). After overnight incubation, cells were isolated via centrifugation, followed by viability staining with Live/Dead Fixable Zombie Aqua dye (BioLegend). The Live/Dead-stained cells were fixed and permeabilized with fixation buffer (BD) and permeabilization/wash buffer (BD), followed by intracellular staining for GzmB (APC anti-human/mouse GzmB recombinant antibody, clone QA16A02, BioLegend). Stained cells were then analyzed for intracellular GzmB expression of tumor cells and T cell cytotoxicity via flow cytometry (Cytek Aurora).

**1.9. Measurements for body weight changes upon systemic nanosensor administration**

Experiments were carried out under the Institutional Animal Care and Use Committee (IACUC) protocol (protocol number: A100281) of the Georgia Institute of Technology. Female C57BL/6 mice (2 to 5 months old, The Jackson Laboratory) received intravenous saline (~150 µL) or nanosensor (140 nM in ~150 µL of saline solution) injection through a tail-vein. The body weight for individual mice was measured every three days up to day 15 post-nanosensor or saline injection.

**1.10. *In vivo* PA tumor imaging following adoptive T cell transfer (ACT)**

Experiments were carried out under the Institutional Animal Care and Use Committee (IACUC) protocol (protocol number: A100281) of the Georgia Institute of Technology. EL4 (1 million cells) or EG7-OVA (1.3 million cells) tumor cells were subcutaneously inoculated into the flank of female C57BL/6 mice (5 to 6 weeks old, The Jackson Laboratory). OT1 T cells (10 million cells) were systematically administrated via the tail-vein injection on day 5 post-tumor inoculation. Mice were anesthetized with isoflurane and positioned on a small animal heating pad to maintain body temperature during ACT. Over the next 48 hours following ACT, all mice received 2 two doses of 100,000 units of IL-2 (Reprotech) intraperitoneally. Tumor volumes were measured by π/6 $\times$ length $\times$ width^2^.

To investigate PA signals from GzmB-activated nanosensors in EL4 or EG7-OVA tumors treated with or without ACT, the GzmB-activated plasmonic nanosensors (140 nM in ~ 150 µL of saline solution) were intravenously injected through a tail-vein on day 6 post-tumor inoculation (or day 1 post-ACT). US/PA imaging was performed before and after the nanosensor injection via Vevo2100/LAZR imaging system equipped with an LZ250 US transducer (center frequency: 21 MHz, bandwidth range: 13-24 MHz, focal depth: 10 mm) integrated with an optical-fiber light delivery system. Laser pulses were generated by a Q-switched Nd:YAG-pumped optical parametric oscillator (OPO) laser (laser fluence: ~10 mJ cm^-2^, pulse duration: 7 ns, pulse repetition frequency: 20 Hz). The PA gain and B-mode gain was set as 40 dB and 18 dB, respectively. Mice were anesthetized with isoflurane and positioned on a small animal heating pad to maintain body temperature during nanosensor injection and imaging. The subcutaneous EL4 or EG7-OVA tumor was covered with ultrasound gel (3B Scientific) to facilitate coupling with US/PA transducer (LZ250). To ensure consistent imaging across sessions, tumors were aligned according to anatomical landmarks and representative cross-sectional areas identified in B-mode US imaging. Due to the slight variations in imaging planes across and within groups, US/PA imaging of tumor-bearing mice was performed with multiple biological replicates (n = 5-7) to ensure reproducibility and consistency. All acquired imaging data were analyzed using VevoLAB5.7.0 software to quantify primary tumor signals based on US images. The in vivo US/PA images were further post-processed in MATLAB (MathWorks, Inc.) using a Gaussian filter to reduce noise and artifacts. Representative tumor images were presented at 700 nm wavelength by segmenting the tumor region according to anatomical information from the ultrasound B-mode image.

**1.11. Tumor dissociation and flow cytometry analysis**

Experiments were carried out under the Institutional Animal Care and Use Committee (IACUC) protocol (protocol number: A100281) of the Georgia Institute of Technology. EL4 (1 million cells) or EG7-OVA (1.3 million cells) tumor cells were subcutaneously inoculated into the flank of female C57BL/6 mice (5 to 6 weeks old, The Jackson Laboratory) or the flank of female B6 CD45.1 mice (B6.SJL-Ptprca Pepcb/BoyJ, 5 to 6 weeks old, The Jackson Laboratory). OT1 T cells (10 million cells) were systematically administrated via the tail-vein injection on day 5 post-tumor inoculation. Over the next 48 hours following ACT, all mice received 2 two doses of 100,000 units of IL-2 (Reprotech) intraperitoneally. On day 2 post-ACT, EL4 or EG7-OVA tumors were isolated, followed by mechanical and enzymatic dissociation with DNAse I (Sigma Aldrich) and collagenase (type IV, Sigma Aldrich). Tumor-infiltrating lymphocytes were isolated using a density gradient with 44% Percoll centrifugation media (Sigma Aldrich) in PPMI 1640, followed by red blood cell lysis (BioLegend). For flow cytometry, cells were treated with Fc blocker (anti-mouse CD16/CD32, Tonbo Biosciences) in cell staining buffer (BioLegend) and stained with surface markers, including CD3 (FITC anti-mouse CD3e, clone 145-2C11, BioLegend), CD45.2 (PE anti-mouse CD45.2, clone 104, BioLegend) and CD8 (PerCP/Cy5.5 anti-mouse CD8, clone 53-6.7, BioLegend), in cell staining buffer (BioLegend). Next, viability staining of surface marker-stained cells with Live/Dead Fixable Zombie Aqua dye (Biolegend) was performed, followed by fixation and permeabilization with fixation buffer (BD) and permeabilization/wash buffer (BD). The fixed, permeabilized cells were stained with GzmB (APC anti-human/mouse GzmB recombinant antibody, clone QA16A02, BioLegend). The stained cells were then analyzed via flow cytometry (Cytek Aurora). All surface-marker antibodies were used for staining dissociated tumor samples at 1:100 dilution from stock concentrations.

**1.12. Software**

All acquired US/PA imaging data were analyzed using VevoLAB 5.7.0 and MATLAB R2023a software. Statistical analyses were performed using GraphPad Prism 8.0.2. Two-tailed unpaired or paired Student’s t-tests were used for comparisons between two groups. One-way ANOVA with Tukey’s post-hoc test was applied for multiple group comparisons, while two-way ANOVA with Sidak’s post-test was used to analyze tumor volume changes with or without ACT. Different numbers of asterisks were assigned to the graphs showing the statistical comparison across different groups (ns: non-significant difference; *: p < 0.05; **: p < 0.01, ***: p < 0.001, ****: p < 0.0001).

**Supplementary Note 2.** Supplementary Figures

**
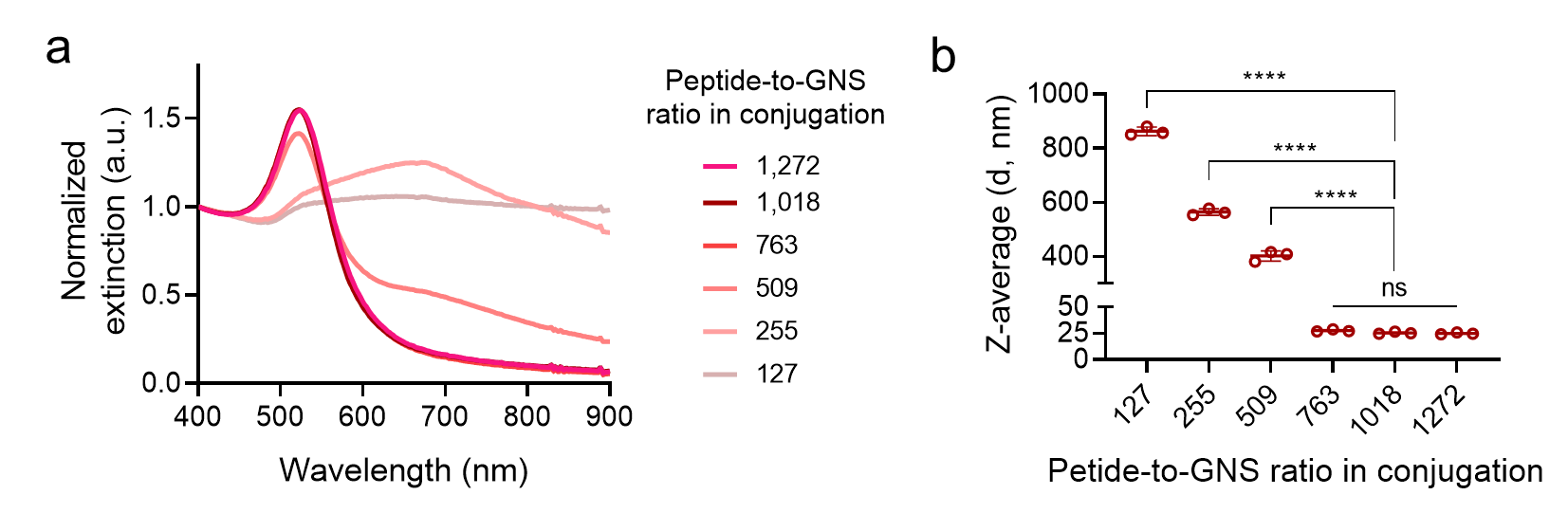
**

**Figure S1 |** a, b) UV-vis-NIR spectra (a) and hydrodynamic diameter (b, n=3) of GNSs functionalized with GzmB-cleavable peptides at different stoichiometric ratio of peptide to GNS. Data are presented as the mean ± standard deviation. The statistical analysis for Figure S1b was conducted using a one-way ANOVA with Tukey post-hoc tests. The statistically significant difference is represented as the asterisk (ns: non-significant, ****: p < 0.0001).


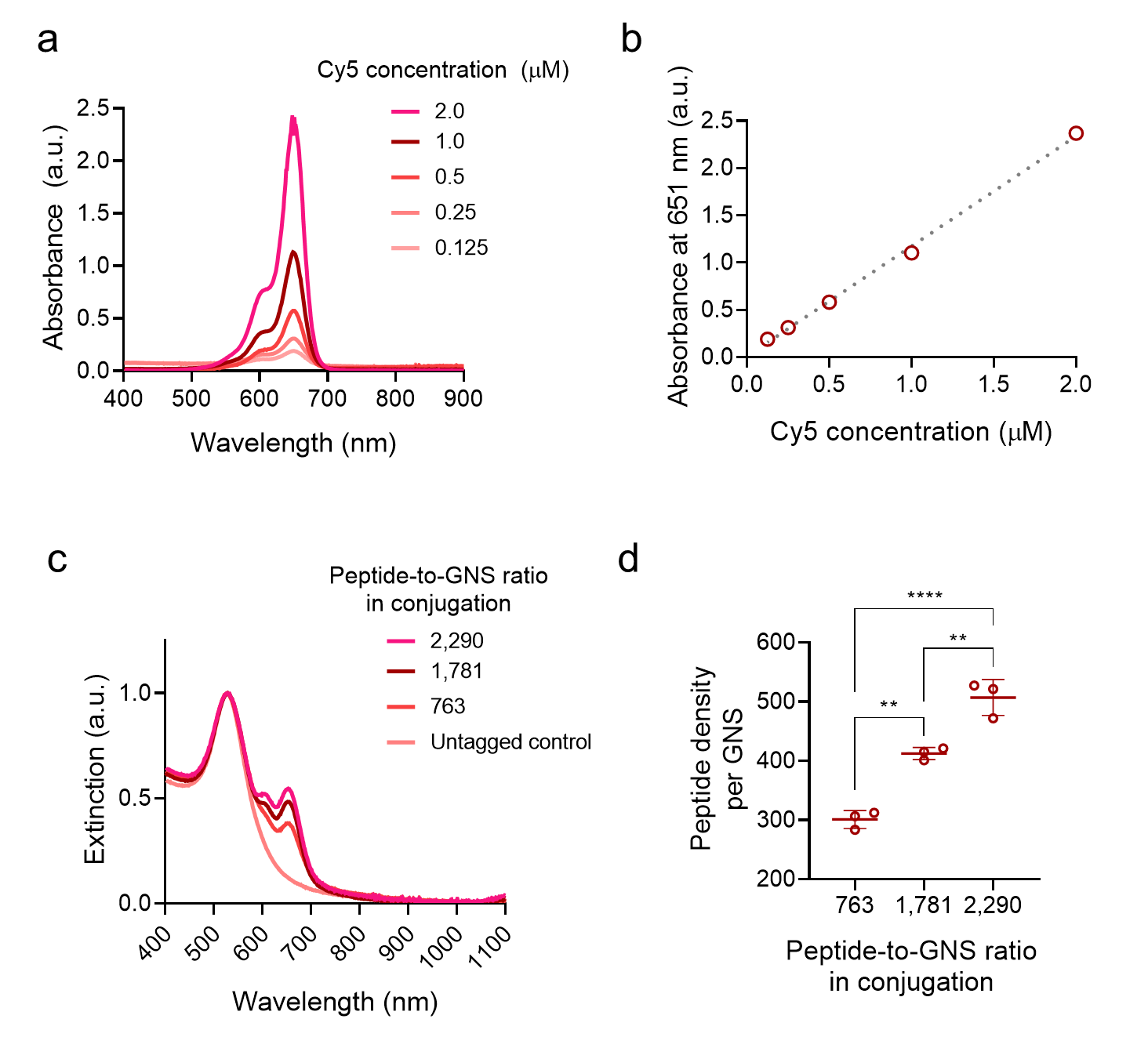


**Figure S2 |** a, b) UV-vis-NIR spectra (a) and absorbance at 651 nm of (b, n=3) of Cy5-NHS ester molecules. c, d) UV-vis-NIR spectra (c) and corresponding peptide density (d, n=3) of GNSs functionalized with Cy5-tagged GzmB cleavable peptides at different peptide-to-GNS ratios. Data are presented as the mean ± standard deviation. The statistical analysis for Figure S2d was conducted using a one-way ANOVA with Tukey post-hoc tests. The statistically significant difference is represented as the asterisk (**: p < 0.01, ****: p < 0.0001).

**Figure S3 |** a, b) Time progression of extinction ratio (700 nm/520 nm) of GzmB-activated plasmonic nanosensors with different peptide densities in the absence of GzmB (n=3). Data are presented as the mean ± standard deviation.

**Figure S4 |** a, b) Time progression of extinction ratio (700 nm/520 nm) of GzmB-activated plasmonic nanosensors upon exposure to GzmB with different concentrations (unit: nM, n=3). Data are presented as the mean ± standard deviation.

**Figure S5 |** Time-dependent changes in normalized absorbance at 700 nm of the GzmB-activated plasmonic nanosensors in the media of stimulated murine cytotoxic T cells, compared to that in the media of unstimulated T cells (n=3). Data are presented as the mean ± standard deviation.


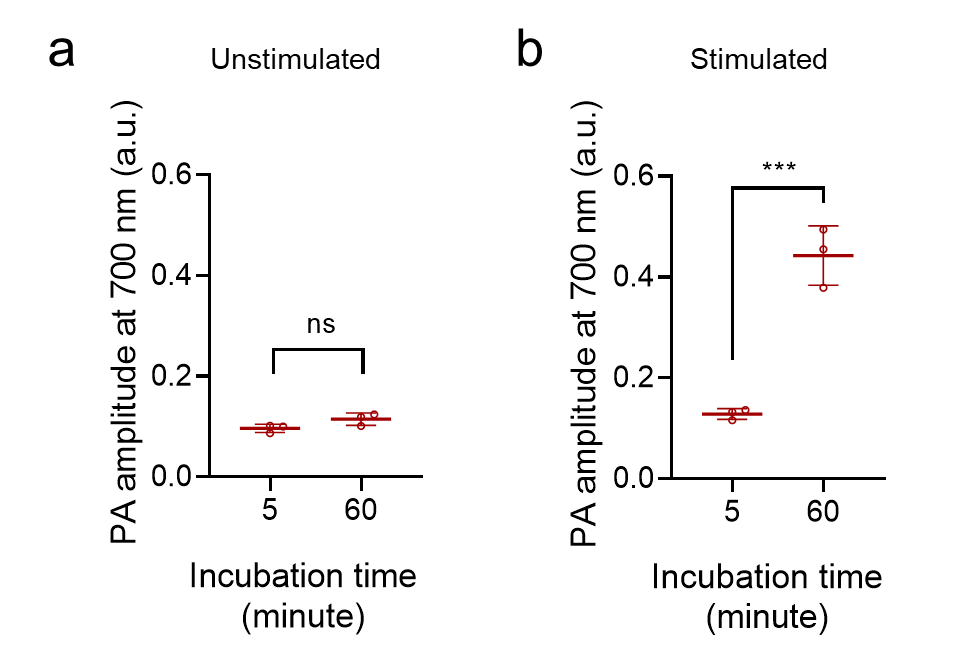


**Figure S6 |** a, b) Comparison of PA responses of the GzmB-activated plasmonic nanosensors in the media of unstimulated (a, n=3) or stimulated (b, n=3) murine CD8+ T cells at different time points post-incubation. Data are presented as the mean ± standard deviation. The statistical analysis was conducted using a two-tailed Student’s t-test. The statistically significant difference is represented as the asterisk (ns: non-significant, ***: p < 0.001).

**Figure S7 |** PA signals from GzmB-activated plasmonic nanosensors within the 700-900 nm spectral range in the media of unstimulated or stimulated murine CD8+ T cells measured 1-hour after incubation (n=3). Data are presented as the mean ± standard deviation.

**Figure S8 |** Time-dependent changes in absorbance at 700 nm of the GzmB-activated plasmonic nanosensors in the co-culture media, normalized to EL4 tumor media (n=4). Data are presented as the mean ± standard deviation.

**Figure S9 |** PA signals from GzmB-activated plasmonic nanosensors within the 700-900 nm spectral range in the co-culture media (n=3). Data are presented as the mean ± standard deviation.

**Figure S10 |** Body weight changes of mice that received saline control or GzmB-activated nanosensors (n=4). Data are presented as the mean ± standard deviation. The statistical analysis was conducted using a two-way ANOVA with Sidak’s post-test. The statistically non-significant difference is represented as ns.


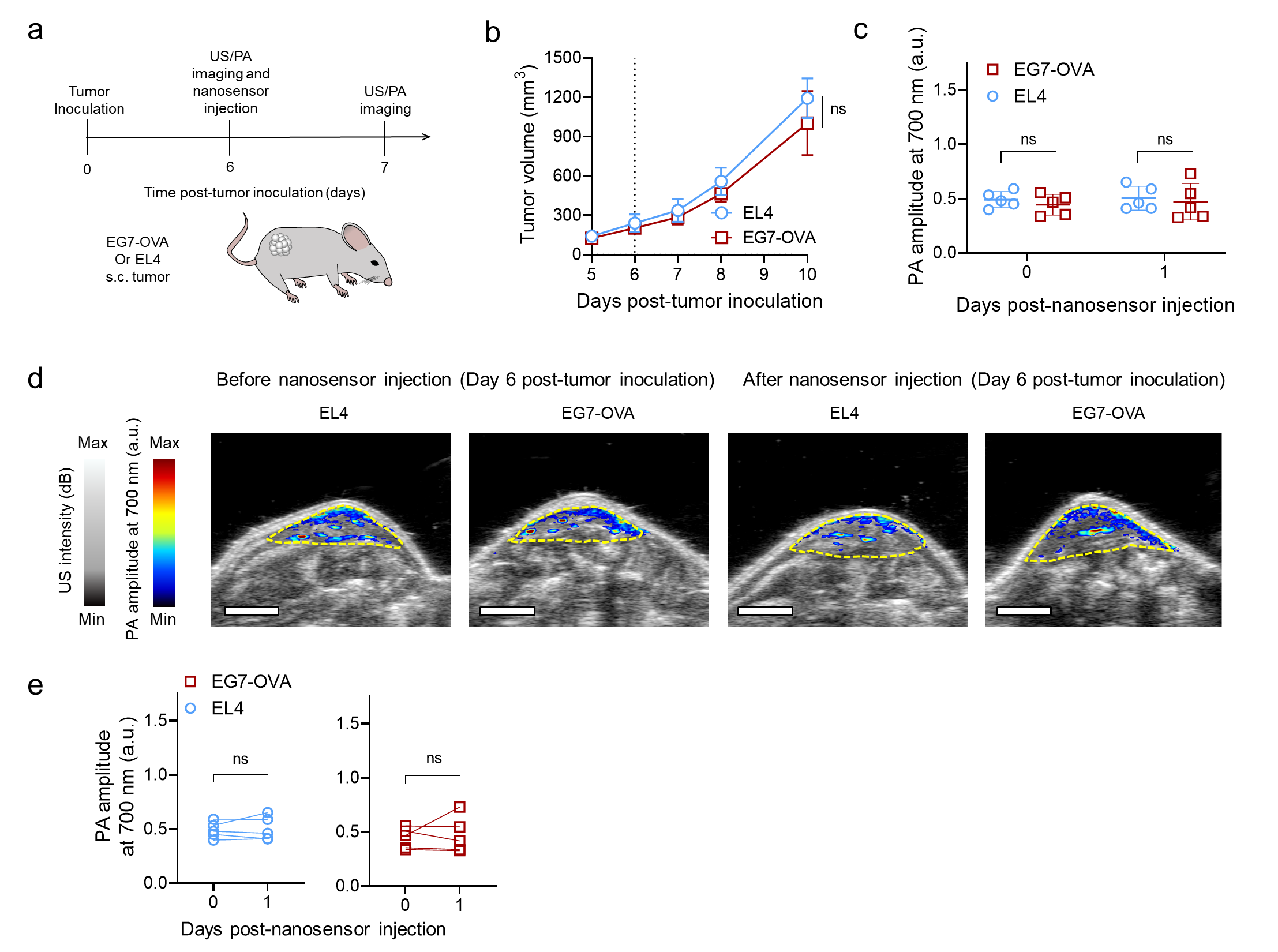


**Figure S11 |** a) Schematic illustration of workflow of US/PA imaging with GzmB-activated nanosensor administration. b) Measured volume changes of EL4 or EG7-OVA tumors in mice treated with nanosensor administration (n=5). c, d) PA responses (c) and US/PA images (d) of EL4 or EG7-OVA tumors in mice treated before and after systemic nanosensor administration (n=5). Scale bars are 4 mm. e) PA signal comparison of EL4 or EG7-OVA tumors in mice before and after nanosensor injection. Data are presented as the mean ± standard deviation. The statistical analysis for Figure S11b was conducted using a two-way ANOVA with Sidak’s post-test. The statistical analysis for Figure S11c was conducted using an unpaired two-tailed Student’s t-test. The statistical analysis for Figure S11d was conducted using a paired two-tailed Student’s t-test. The statistically non-significant difference is represented as ns.

Note: US/PA imaging in Figure S11 was performed in tumor-bearing mice without ACT, whereas Figure 4 represents US/PA imaging in mice with ACT. In both experiments, nanosensors were systematically injected into tumor-bearing mice on day 6 post-tumor inoculation, serving as the reference point for imaging timelines.

**
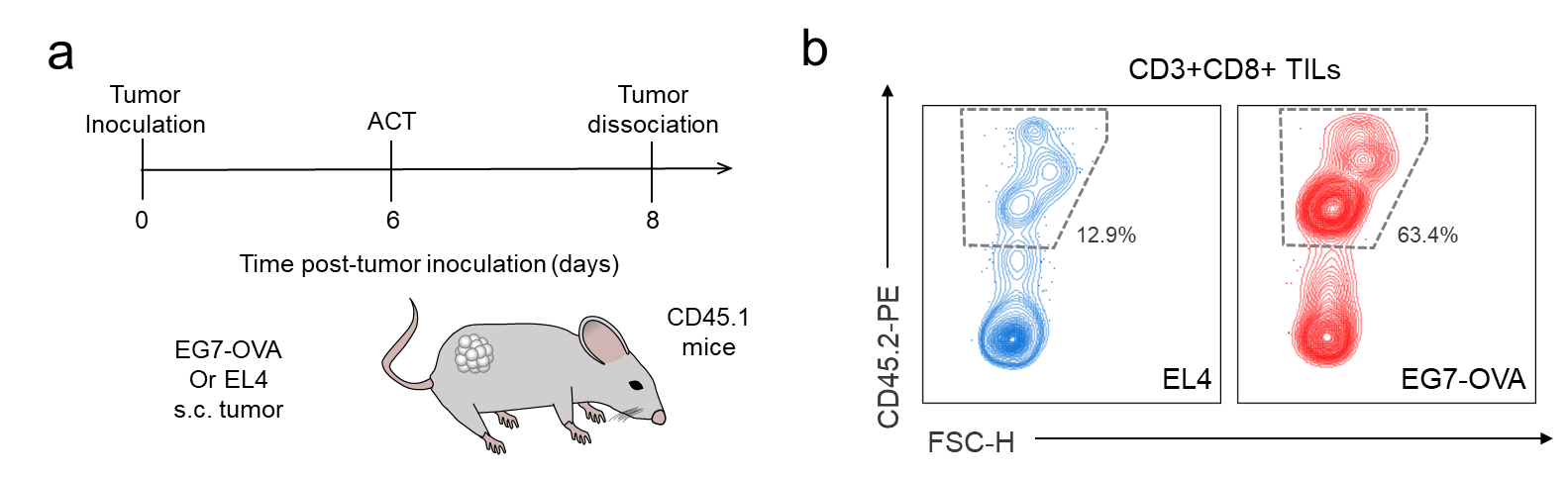
**

**Figure S12 |** a) Schematic illustration of workflow of ACT and tumor dissociation for flow cytometry in a CD45.1 mouse model. b) Representative flow plots of the percentage of transferred OT1 T cells across CD3+CD8+ T cells in EL4 or EG7-OVA tumors.

**Figure S13 |** Individual spider plots of volume changes of EL4 or EG7-OVA tumors following adoptive cell transfer.


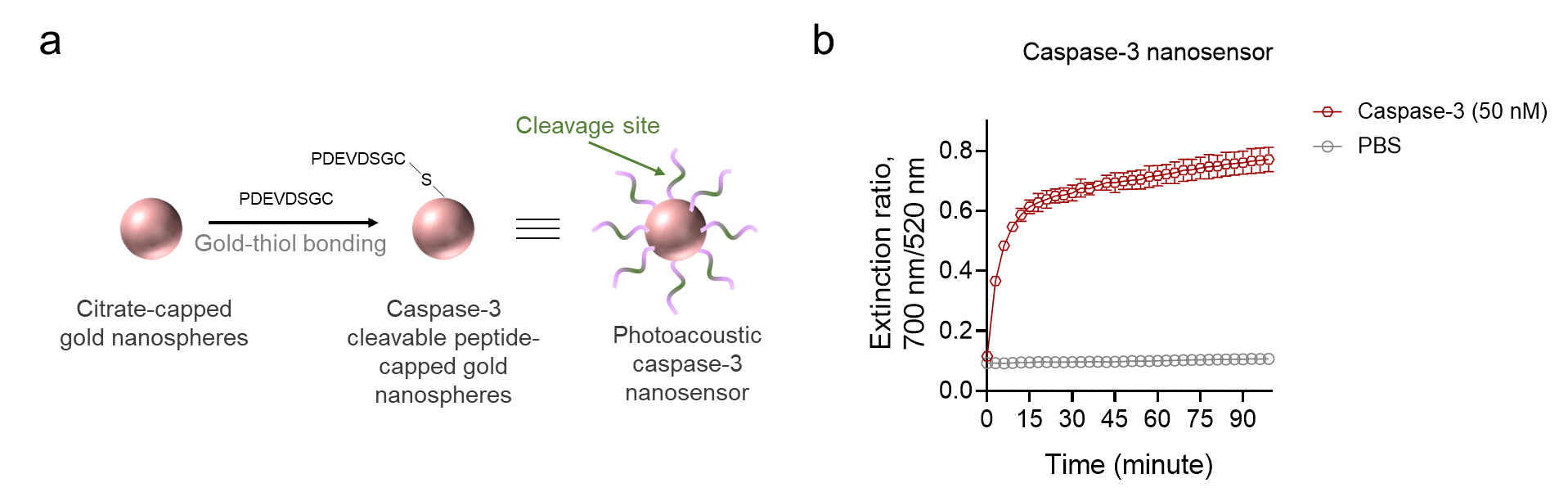


**Figure S14 |** a) Schematic illustration of the synthesis of Cas-3-activated plasmonic nanosensors. b) Time progression of extinction ratio (700 nm/520 nm) of the nanosensors in the presence or absence of 50 nM Cas-3 (n=3). Data are presented as the mean ± standard deviation.

**References**

[1] J. Turkevich, P. C. Stevenson, J. Hillier, *Discuss. Faraday Soc.* 1951, *11*, 55.

[2] J. Kimling, M. Maier, B. Okenve, V. Kotaidis, H. Ballot, A. Plech, *J. Phys. Chem. B* 2006, *110*, 15700.

[3] P. B. Johnson, R. W. Christy, *Phys. Rev. B* 1972, *6*, 4370.
